# Assessing the impact of genomic selection against hip dysplasia in the Labrador Retriever dog

**DOI:** 10.1111/jbg.12056

**Published:** 2013-10-18

**Authors:** E Sánchez-Molano, JA Woolliams, SC Blott, P Wiener

**Affiliations:** 1 The Roslin Institute and Royal (Dick) School of Veterinary Studies, University of EdinburghEdinburgh, UK; 2 Kennel Club Genetics Centre at the Animal Health TrustNewmarket, UK

**Keywords:** genomic selection, canine hip dysplasia, Labrador Retriever

## Abstract

Many purebred dogs exhibit a higher prevalence of inherited diseases compared with non-purebred dogs. One of the most popular breeds in the UK is the Labrador Retriever, which has a high prevalence of hip dysplasia resulting in high costs for surgical operations and impaired animal welfare. Considering the many complications of highly managed populations, mainly due to breeder's conventions and the resulting population structure, is of great importance for the proper development of a strategy against the disease. In this study, we have compared the utilities and performances of both genomic and phenotypic selection against hip dysplasia in a simulated population with the characteristics of the British Veterinary Association and Kennel Club (BVA/KC) hip dysplasia scheme. The results confirm the potential benefits of genomic selection by showing a moderate increase of 1.15-fold (assuming a realistic accuracy of *r*^2^ = 0.5) in response to selection due to the higher accuracy (between 0.96- and 1.32-fold, considering 0.35 ≤ *r*^2^ ≤ 0.7) and more than a threefold increase when all the offspring in each litter are tested (between 3.25- and 4.55-fold, again considering 0.35 ≤ *r*^2^ ≤ 0.7).

## Introduction

Intensive breeding programmes with the objective of maintaining standard dog breeds and/or generating championship-calibre animals have resulted in a number of inherited disorders due to inbreeding and associations between selected and disease traits. The prevalence of some diseases such as syringomyelia (with 25–70% prevalence in the King Charles Cavalier Spaniel; Parker *et al*. 2011) and hip dysplasia (with 25–40% prevalence in UK Labrador Retrievers; reviewed by Coopman *et al*. 2008) is extremely high in certain breeds (McGreevy & Nicholas 1999), and surgery or other invasive treatments are often necessary to combat these diseases. As a result of increased concern for animal welfare and adverse media attention focusing on these problems (Lewis *et al*. 2010), several modifications have recently been applied to some breeding programs. As many of these disorders are complex diseases resulting from the combination of many genes and environmental factors, simple Mendelian genetic tests cannot be used to remove affected or carrier animals from the gene pool, and thus, other strategies must be used.

Hip dysplasia is an example of a complex disorder; it has a relatively high heritability, *h*^*2*^ = 0.35 (Lewis *et al*. 2010) and high prevalence in breeds of large size, including the Labrador Retriever, the German Shepherd and the Golden Retriever, which are amongst the 10 most popular UK breeds (BVA & KC 2011). This disease entails an abnormal formation of the hip socket, resulting in coxofemoral (hip) joint laxity with a deformed femur head and/or acetabulum, which causes abrasion when the joint moves. Several pathologies derive directly from the disease (exostosis, cartilage erosions, lameness and osteoarthritis), whereas others are indirect consequences of the anomalous movements made by the dog to avoid pain such as spinal or stifle problems (Lipowitz & Newton 1985).

Scoring of radiographs is traditionally used to detect and evaluate hip dysplasia in the UK (Willis 1997), assessing nine elements in each joint and summing them for both. This type of scoring was established in 1984 by the British Veterinary Association (BVA) and the UK Kennel Club (KC) and is also used in Australia and New Zealand. However, this scoring method is invasive and implies a risk to the dog either from the use of anaesthesia or possible dislocation or damage to the hip joint, as it requires the dog to be positioned on its back with its hind legs extended caudally (Riser *et al*. 1985). Furthermore, the use of the scoring systems in selection programmes against hip dysplasia differs amongst countries (Lewis *et al*. 2010) and also amongst breeds. As an example of the latter, enforced threshold values for the breeding stock are applied in some of the 71 involved breeds in Finland, whilst for other breeds, only the screening is mandatory (Leppanen & Saloniemi 1999). In the UK, the BVA/KC scheme involves 126 breeds, with recommendations to use the breed mean as a threshold (BVA 2012) although this is not mandatory.

In general, reports on the response to current phenotypic selection schemes in several breeds showed mixed results, with a small improvement against the disease in some cases (Malm *et al*. 2008; Lewis *et al*. 2010) or no discernible improvement in others (Willis 1997; Leppanen & Saloniemi 1999). These results may be due to various factors, for example, no attempt is made to remove systematic environmental influences (e.g. age) from the raw phenotypes or to reduce random environmental variation by including information from relatives.

Given these factors, an alternative strategy to phenotypic selection is the use of genomic selection, which has been implemented in several livestock populations and shows very good results in comparison with classical selection methods (Hayes *et al*. 2009). In this strategy, a DNA test provides information on dense marker genotypes in a training population of phenotypically scored animals, providing a subset of markers in linkage disequilibrium with genes associated with the disease (Meuwissen *et al*. 2001). These markers are used to compute genomic estimates of the true breeding values of the animals (genomic breeding values, GEBVs) and could be used for selection in a new sample within the same breed, with no need for phenotypic information, although re-estimates of the marker effects may be needed every few generations. The potential advantage of genomic selection over classical phenotypic selection against hip dysplasia is that GEBVs would be corrected for environmental influences. Whilst this is also true for BLUP-EBVs (Lewis *et al*. 2013), the accuracy of selection should increase over time as the number of animals in the training population increases. Moreover, GEBVs distinguish amongst unrecorded littermates, thus allowing selection within families at a young age and so increasing response without introducing severe penalties on the rate of inbreeding (Daetwyler *et al*. 2007).

Therefore, the purpose of this study is to compare the performance of genomic selection against the benchmark of phenotypic selection when used to select against hip dysplasia. This is achieved by simulating a population and recording scheme similar to the Labrador Retrievers and the BVA/KC scheme in the UK, in which many complications of this highly managed population are considered.

## Materials and methods

### Characteristics for modelling population

#### BVA/KC regulations and hip-scoring scheme

KC (2012) has regulations governing the age of the parents of new registrations. A litter will not be registered if the dam has reached the age of 8 years at the date of whelping or if the dam has previously had 4 litters registered, although before 2012, the maximum number of litters allowed was 6. There are no restrictions on the age of sires. No parent–offspring or full-sib matings are allowed. It is strongly recommended not to mate a female until its second oestrous cycle (between 1.6 and 2 years old).

The BVA/KC hip-scoring scheme is voluntary with the participation of approximately 8–10% of all Labrador Retrievers registered in a year (∼4000 dogs p.a. based on 45,779 registrations in 2005). Animals must be 1 year or older at scoring to assure skeletal maturity, but there is no upper age limit. Data from the hip score scheme obtained between 1996 and 2006 shows that 90% of the available information comes from animals between 1 and 4 years old, with a higher number of females than males (75% versus 25%). Hip score values taken from radiographs of dogs between 1 and 3 years old reveal a highly skewed distribution with mean 13.22, median 10 and coefficient of skewness of 3.39. Given the skewness of this distribution, the use of a logarithmic transformed scale has been recommended, such that the transformed hip value (TH) is computed as *log*_*e*_(1 + *HS*) where HS is the actual hip score value (Lewis *et al*. 2010).

Although the BVA/KC recommendation is to breed animals below the phenotypic mean (Lewis *et al*. 2010), many breeding decisions tend to be made prior to scoring as, according to social preferences, most breeders will sell part of each litter when they are puppies, keeping only a small proportion (selected according to other traits) for potential breeding. Thus, the current selection scheme has resulted in a selection intensity equivalent to selecting the above the 85th percentile of the TH distribution and avoiding only the worst 15% of animals (Lewis *et al*. 2010).

**Figure 1:**
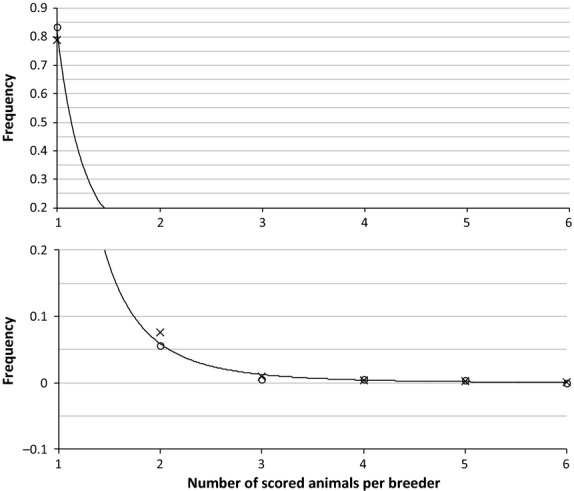
Frequency of the number of scored males (circles) and females (crosses) per breeder in the data set. Fitted curve (thin line) corresponds to equation *y* = 0.84*x*^−3.92^, fitted by regression.

**Figure 2:**
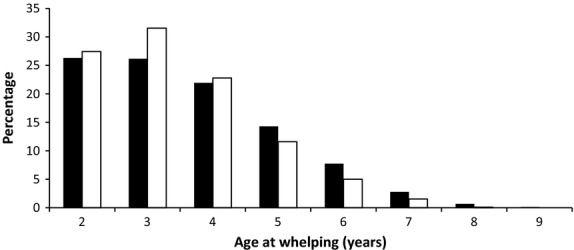
Age at whelping of sires (black) and dams (white). Scales are given in percentages, and age was rounded to the closest integer.

The analysis of the BVA/KC data set shows that the number of scored animals per breeder follows a probability distribution as observed in Figure 1, with approximately 90% of breeders scoring one or two animals per litter. The distribution of the age at whelping (Figure 2) shows a similar pattern for both sexes, with the youngest being 2 years old and the oldest being 7 (dams) or 8 (sires), such that fewer than 15% of animals are older than 5 years old when mated. These results are in agreement with the BVA/KC regulations and the recommendations mentioned above.

**Figure 3:**
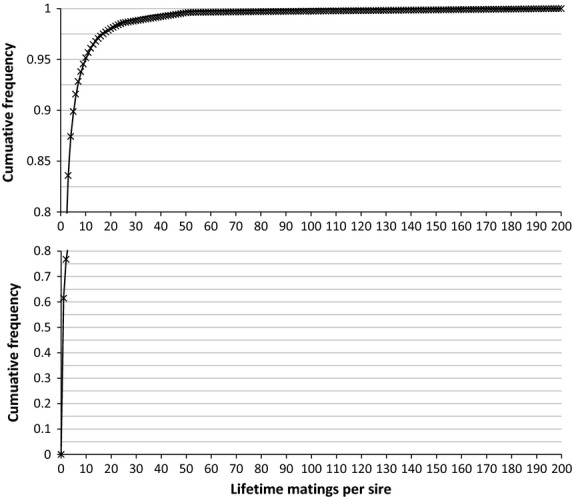
Cumulative density function observed (crosses) and expected (thin line) for the number of matings per male lifetime in the data set. Expected probability distribution was fitted by regression to *y* = 0.63*x*^−2.01^ up to a value of 25 matings, whereas for 26–50 matings, probability was equal to 4 × 10^−3^, and for 51–200 matings, probability was equal to 2.3 × 10^−5^.

**Figure 4:**
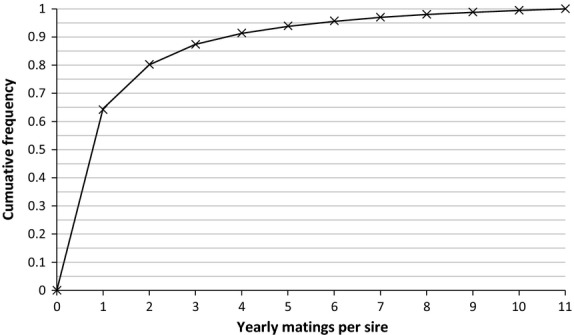
Cumulative density function observed (crosses) and expected (thin line) for the number of matings per male and year in the data set. Expected probability distribution was fitted by regression to *y* = 0.63*x*^−1.98^ up to a value of 11 matings. Expected values were adjusted to reach a maximum cumulative proportion of 1.

The existence of a few popular males in the BVA/KC data set is revealed in the cumulative density functions of the lifetime and annual number of matings per male (Figures 3 and 4, respectively). Less than 5% of the sires produce 12 litters or more across their lifetime (which would be expected to produce 72 puppies, assuming an average litter size of 6), similar to the situation amongst several other UK dog breeds. This is consistent with the results of Calboli *et al*. (2008) who observed 5% of sires as ‘popular’ with more than 100 recorded offspring in the full pedigree of UK Labrador Retrievers.

#### Theoretical background of genomic selection

The theoretical model for the simulation of a genomic predictor described below allows for the assumption of different accuracies, but to simplify the analysis, it does not consider any need to update marker effects every few generations due to the reduction in accuracy caused by recombination (Wolc *et al*. 2011). The effects of these assumptions will be discussed below.

For a given trait with additive gene effects, the phenotype *y* of an individual *i* can be decomposed into two main components: the individual's true breeding value TBV_i_ (which represents the sum of gene effects of the animal) and the individual's environmental deviation e_i_. When adjusting the population mean to 0, both components can be transformed to follow normal distributions with their corresponding additive genetic 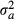 and environmental 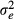 variances:


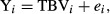



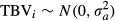



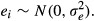


To estimate the true breeding value of an individual using high-density marker data, TBV_i_ can be decomposed again into two components: the individual's genomic estimated breeding value GEBV_i_ (the variance captured by the markers; Dekkers 2007) and the individual's prediction error PE_i_, as markers will not capture all the variance associated with the trait. Thus, GEBV estimates are associated with a prediction accuracy (the correlation between GEBV and TBV), which measures the strength of the relationship between GEBV and TBV. If both components of TBV are assumed to follow normal distributions:


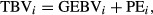



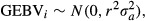



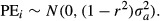


Following classical theory (e.g. Woolliams 2007), the true breeding value of the offspring TBV_off_ can be computed as the average of the parental TBVs plus a Mendelian sampling term MS (deviation of the offspring value from the parent mean due to sampling of parental alleles). This MS term follows a normal distribution 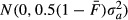, where 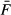 is the average inbreeding coefficient of the parents. We can decompose TBV_off_ into its two components:


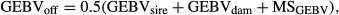



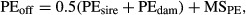


Where


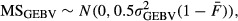



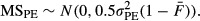


#### Male popularity and social structure

Popularity effects occur when an animal with fashionable attributes, sometimes morphological, is bred repeatedly. Whilst a popular male can sire a large number of litters, the effect of a popular dam is more limited. Thus, male puppies of popular sires tend to be popular amongst breeders, and their use within a breed tends to generate a population structure composed of sublineages derived from these popular sires.

Popularity is considered here as a fashionable attribute of a male leading to different numbers of matings per sire. In a pedigree, popularity acts at two different levels: (i) within an age cohort and across years, popular male candidates will have a greater probability than others to be chosen for breeding at each breeding opportunity; and (ii) within each annual breeding pool, popular sires will be mated more frequently than others.

A simple approximation is to assume that a sire's popularity can be modelled as a continuous trait (Pop) distributed *N*(0,1), with non-genetic inheritance, for example based on reputation, that is assumed to account for transgenerational effects. Here, this continuous trait was inherited by male offspring only through the sire as:


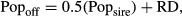


Where Pop_*off*_ and Pop_*sire*_ are the popularity values of the male offspring and the sire, and RD is a random deviation normally distributed with mean 0 and variance 0.75. The value of 0.75 was chosen to maintain the population variance of Pop, assuming that 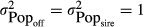 if there was no selection, as 
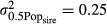
. The use of this underlying trait for mating purposes is described below.

### Computer simulations

A simulated population using FORTRAN was modelled on the characteristics described above, assuming that all animals remain within a hip improvement scheme and no external animals enter the scheme. An overview of the scheme simulated involves the following: for each litter born in the breeding population, typically 1/3 will proceed to become breeding candidates and these are chosen at a young age prior to phenotypic scoring, but after genotyping if using genomic testing. For phenotypic selection, it is assumed that all candidates will ultimately acquire a phenotypic record before any mating. Each year, 4000 puppies become candidates (75% females and 25% males) in accordance with the scale of current recording. The parents for breeding each year are chosen from amongst the candidates, first to establish the age distribution and second from above the 85% percentile for the index of hip score used in scheme. The selection index was either a dog's own phenotype (phenotypic selection) or its GEBV (genomic selection). Therefore, in phenotypic selection, selection against hip dysplasia occurs only after being chosen as a candidate but before proceeding to be a parent, whereas for *GEBV*, it occurs before proceeding to be a candidate and again before proceeding to be a parent.

#### Testing and recording

For each litter, *n* puppies were chosen to become breeding candidates where *n* was 1/3 of the litter rounded to the nearest integer, that is, *n* = 2 animals from litters of 5 or 7. In phenotypic selection, these *n* puppies were chosen at random to be scored later, at the appropriate age, although theoretical scenarios for phenotypic selection where a higher percentage was retained for testing were also simulated for comparison. In the case of genomic selection, given that a DNA test can be applied at birth, more than these *n* puppies could be scored, such that an initial screen can be applied. To simulate this, a proportion of puppies (33, 50 or 100% of the litter, rounded to the closest integer) were chosen at random, scored and then ranked, before selecting the *n* best ranked ones as breeding candidates. When available, at least one female offspring from each litter was chosen as a candidate for breeding, at random in the case of phenotypic selection or as the best scored female in the case of genomic selection.

#### Base population

Each of the age cohorts required for establishing the base population had 4000 animals (75% females and 25% males), and at year 0, all animals in the base population were assumed to be 2 years old. All animals in the base were considered to be unrelated. For each animal, GEBV, PE, TBV and phenotype for TH were computed according to the theory described above, assuming a phenotypic variance of the trait 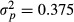 and a heritability *h*^2^ = 0.35 (Lewis *et al*. 2010) and squared accuracies (*r*^2^) of 0.99, 0.7, 0.5 and 0.35. The underlying popularity for each male in the base population was sampled from a normal distribution *N*(0,1). Under phenotypic selection, *r*^2^ is equal to the heritability of the trait.

#### Selection of candidates to become parents in each year

This proceeded in two steps, the first to obtain the age profile, the second to apply selection against hip dysplasia. Each year, a pool of 4000 potential parents was sampled from amongst all candidates, 75% females and 25% males. Two possible schemes were simulated: overlapping and discrete generations. For discrete generations, animals were all 2 years old. For overlapping generations, this pool was filled with candidates of different ages, from 2 to 7 years for females and from 2 to 8 years for males according to the distribution shown in Figure 2 and chosen at random within their age cohort and sex. For females, there was an additional caveat in that they had had fewer than the maximum number of litters per lifetime (4 litters). With overlapping generations, popularity across years was considered in recruitment to the pool. In each year of breeding, within each contributing cohort, all male candidates were ranked by their underlying popularity value and a discrete popularity value was assigned to each male, according to its rank, from the probability distribution corresponding to Figure 3. These discrete popularity values were used to form a probability distribution for sampling the required number of males from that cohort into the pool of potential parents for that year. In this way, popular males had a higher probability of being recruited to the pool.

Following the formation of the annual pool of potential parents, truncation selection was then applied to the pool, ranking all animals by the index, either GEBV (genomic selection) or phenotype (phenotypic selection) and with only the best 85% (the lowest of index values) being allowed to proceed as potential sires and dams.

Matings for that year were then carried out (as described above in ‘Testing and recording’) in sufficient number to produce 4000 candidates. For each mating, a dam was chosen at random, and without replacement, from the pool of potential dams. The potential sires were ranked regardless of age, and then, an expected number of matings was allocated, according to their popularity rank and following the probability distribution described in Figure 4. This expected number of matings was used to form a probability distribution for the sires. For each mating, the sire was then drawn at random, and with replacement, from this distribution. With this sampling, dams only had 1 litter per year but sires might have multiple litters per year, according to their popularity. Mating between close relatives (parent–offspring and full-sib mating) was prevented. For each pair, litter size was sampled from a truncated *N*(6.72, 5.29) distribution (Hare & Leighton 2006), rounding to the closest integer and computing the trait values of the offspring according to the theory described above.

#### Output from simulations

Each scenario encompassed 30 years and was replicated 50 times, with results averaged across replicates. For each set of parameters, the mean and variances for GEBV, PE, TBV and phenotype across all the offspring (age 0) were computed each year, and the inbreeding coefficient of each individual was also estimated from genealogical relationships and averaged for each year. The rates of increase per year for all traits and inbreeding were computed using linear regression over the last 20 years to avoid the mixing effects of the first generations.

### Population structure and additional simulations

Population structure could be an important element to consider when simulating a population. As one of the main causes of population structure is the lack of random mating derived from mating preferences in each year, the departure from random mating (deviation from Hardy–Weinberg proportions) was measured in both the BVA/KC data set (using a pedigree with 5 generations over the data set animals, as in Lewis *et al*. 2010) and simulated pedigrees assuming:


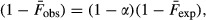


where 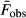 is the average inbreeding coefficient observed in a set of *k* animals, α is the measure of departure from random mating and 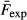 is the average inbreeding coefficient expected from randomly mating the parents of the *k* animals.

Additional simulations under the overlapping model were also performed to study the effects of a lower population size (expected to lead to greater inbreeding) or a higher popularity inheritance (expected to create stronger paternal lines within the population). Thus, three other scenarios were simulated with reduced population sizes (2000, 300 and 80 candidates per year), and two new scenarios were developed by assuming higher popularity inheritances: (i) 0.9 of the sire value plus a random deviation from *N*(0,0.19) and (ii) 0.99 of the sire value plus a random deviation from *N*(0, 0.02).

## Results

The central aim of this study was to compare the performances of genomic and phenotypic selection schemes against hip dysplasia using stochastic simulations and assuming two main models: discrete and overlapping generations. The number of parents needed per year to maintain a population with a constant number of 1000 male and 3000 female candidates per year was approximately 670 sires and 1945 dams with scored offspring, similar for both discrete and overlapping models. However, the models differed in the replacement rate such that all parents were replaced each year for the discrete model, whereas in the overlapping model, approximately 750 males and 2342 females were replaced each year with animals not previously mated. Thus, the results of the overlapping model agreed with the low average number of matings per sire observed in the real data set (approximately 1.2 matings per animal), providing an average age of first litter of 3.32 years for sires and 3.09 years for dams.

Tables 1 and 2 show the average rates of genetic progress (*Δ*TBV) when selecting the best 85% of the population for both discrete and overlapping models, respectively, under various selection schemes and assuming that all breeders used either phenotypic or genomic selection. Values shown are negative because a lower TH value (and HS score) indicates a lower disease severity.

**Table 1:** Scenarios with discrete generations: Rate of genetic gain *ΔG* and inbreeding *ΔF* per year and generation per type of selection (*T*), with phenotypic *P* or genomic *G* selection, squared accuracy *r*^2^, percentage of tested animals per litter *Test* and generation interval *L* (average age of animals in the breeding pool). Rates are averaged over the last 20 years and shown with standard errors. The number of replicates (50) was the same for all cases

			Per year			Per generation	
					
T	*r*^2^	Test, %	ΔG	ΔF (×10^−4^)	L	ΔG	ΔF (×10^−4^)
P	*h*^2^	33	−0.0266 ± 0.0002	2.77 ± 0.02	2	−0.05	5.54
		50	−0.0444 ± 0.0002	2.73 ± 0.02	2	−0.09	5.46
		100	−0.0723 ± 0.0002	2.71 ± 0.02	2	−0.15	5.42
G	0.35	33	−0.0246 ± 0.0002	2.78 ± 0.02	2	−0.05	5.56
		50	−0.0464 ± 0.0002	2.77 ± 0.02	2	−0.09	5.55
		100	−0.0803 ± 0.0002	2.69 ± 0.01	2	−0.16	5.39
	0.5	33	−0.0293 ± 0.0002	2.78 ± 0.02	2	−0.06	5.56
		50	−0.0557 ± 0.0002	2.75 ± 0.02	2	−0.11	5.50
		100	−0.0962 ± 0.0002	2.71 ± 0.02	2	−0.19	5.42
	0.7	33	−0.0348 ± 0.0001	2.81 ± 0.02	2	−0.07	5.62
		50	−0.0662 ± 0.0001	2.73 ± 0.01	2	−0.13	5.46
		100	−0.1137 ± 0.0001	2.70 ± 0.02	2	−0.23	5.40
	0.99	33	−0.0412 ± 0.0001	2.81 ± 0.02	2	−0.08	5.62
		50	−0.0787 ± 0.0001	2.74 ± 0.02	2	−0.16	5.47
		100	−0.1350 ± 0.0001	2.70 ± 0.02	2	−0.27	5.40

**Table 2:** Scenarios with overlapping generations: Rate of genetic gain *ΔG* and inbreeding *ΔF* per year and per generation per type of selection (*T*), with phenotypic *P* or genomic *G* selection, squared accuracy *r*^2^, percentage of tested animals per litter *Test* and generation interval *L* (average age of animals in the breeding pool). Rates are averaged over the last 20 years and shown with standard errors. The number of replicates (50) was the same for all cases

			Per year			Per generation	
					
T	*r*^2^	Test, %	ΔG	ΔF (×10^−4^)	L	ΔG	ΔF (×10^−4^)
P	*h*^2^	33	−0.0147 ± 0.0002	2.61 ± 0.06	3.46	−0.05	9.02
		50	−0.0249 ± 0.0002	2.48 ± 0.03	3.46	−0.07	8.58
		100	−0.0407 ± 0.0002	2.52 ± 0.04	3.46	−0.14	8.70
G	0.35	33	−0.0142 ± 0.0002	2.64 ± 0.04	3.44	−0.05	9.08
		50	−0.0273 ± 0.0002	2.61 ± 0.05	3.39	−0.09	8.86
		100	−0.0478 ± 0.0002	2.56 ± 0.05	3.28	−0.16	8.40
	0.5	33	−0.0168 ± 0.0002	2.60 ± 0.04	3.44	−0.06	8.95
		50	−0.0326 ± 0.0002	2.58 ± 0.04	3.39	−0.11	8.76
		100	−0.0567 ± 0.0002	2.49 ± 0.04	3.28	−0.19	8.17
	0.7	33	−0.0195 ± 0.0002	2.63 ± 0.05	3.44	−0.07	9.03
		50	−0.0381 ± 0.0002	2.52 ± 0.04	3.39	−0.13	8.54
		100	−0.0669 ± 0.0002	2.55 ± 0.05	3.28	−0.22	8.35
	0.99	33	−0.0231 ± 0.0001	2.62 ± 0.04	3.44	−0.08	9.00
		50	−0.0452 ± 0.0002	2.59 ± 0.04	3.39	−0.15	8.76
		100	−0.0798 ± 0.0001	2.54 ± 0.05	3.28	−0.26	8.35

For a given percentage of animals tested per litter, the magnitude of the response to phenotypic selection was lower than that observed for genomic selection, except for *r*^2^ = *h*^2^ = 0.35 with 33% scored as there was no difference in accuracy and no opportunity for early selection within litters to obtain significant benefit. With genomic selection, the response increased as *r*^2^ increased (by 2/3 between *r*^2^ = 0.35 and *r*^2^ = 0.99). However, the greater change in response for both types of selection derived from scoring a larger proportion of the litter, which led to nearly a twofold increase between testing 33% of the litter and testing 50% and to more than a threefold increase when testing the whole litter. Thus, genomic selection was clearly superior, as the best response that could be achieved under phenotypic selection (keeping 100% of each litter to scoring age) was only slightly greater than that achieved under genomic selection with *r*^2^ = 0.7 and testing 50% of each litter.

**Figure 5:**
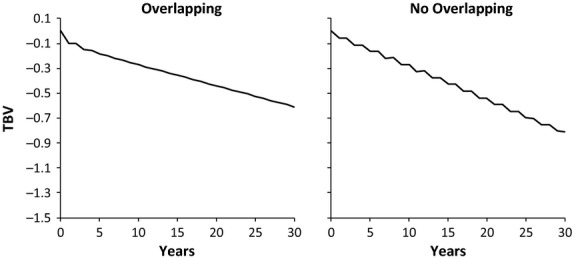
Evolution of the genetic progress per year in the overlapping (left) and non-overlapping scheme (right) for genomic selection with *r*^2^ = 0.5 and testing 33% of each litter.

The discrete model was mainly implemented for illustrative purposes as it is a theoretical construct. Although the genetic progress per generation is similar for both models, the shorter generation interval of the discrete model leads to a higher number of generations at the end of the 30 years, thus resulting in a better final response. However, the main difference between discrete and overlapping generations is the ‘step’ effect that can be observed in Figure 5, where the absence of overlapping creates a situation where the population progresses in generational blocks. It can be observed in Figure 5 that the overlapping model takes approximately 5 years (1–2 generations) to reach steady rates of progress due to the mixing of age blocks in the first years although, as stated in Materials and Methods, we have considered only the last 20 years to compute the rates of progress and inbreeding, to allow rates to stabilize.

**Figure 6:**
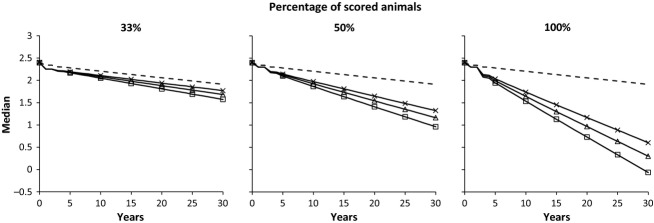
Annual reduction in the TH median selecting the best 85% for the transformed hip score for three percentages of scored animals per litter (33%, 50% and 100%) under an overlapping generations scheme. The dashed line is classical phenotypic selection, whilst solid lines are genomic selection with *r*^2^ = 0.99 (squares), *r*^2^ = 0.7 (triangles) and *r*^2^ = 0.5 (crosses).

The performance of both types of selection against hip dysplasia can be also illustrated by how the TH median changes across years when all breeders apply selection (Figure 6). For the current phenotypic selection (testing 33% of each litter), it would take approximately 12 generations of selection (41 years) to reduce the current TH median by 25%, whereas for genomic selection (with *r*^*2*^ = 0.7), it would take approximately 5 generations of selection (17 years) to achieve the same reduction when scoring 50% of each litter and even less time (6–8 years) when scoring 100%. A reduction of 25% in the TH median corresponds to a 50% reduction in the median of the untransformed hip score.

The rates of inbreeding obtained in our simulated scenarios with overlapping generations were on the order of 9 × 10^−4^ per generation, similar for all scenarios and also similar to that obtained in the BVA/KC data set (1 × 10^−3^ per generation). To study the possible existence of population structure in the BVA/KC pedigree, we investigated the level of random mating amongst individuals, comparing it with the simulated scenarios. The average inbreeding coefficient of the animals born in 2006 showed a low tendency of animals mating with relatives (α = 3%). Regarding our simulations, the average inbreeding coefficient for the last year led to α = 0.02%, also indicating that mating between relatives was rare.

The additional simulated scenarios developed to study the effects of population structure (high popularity inheritance) or inbreeding (low population sizes) led to increases in inbreeding compared with the original scenarios. For a reasonable genomic selection scenario (*r*^2^ = 0.5 and 50% of scored animals), the rate of inbreeding observed for both cases with higher popularity inheritance was 4.4 × 10^−4^ (a 1.7-fold increase), whereas for the low population size, the rates of inbreeding observed were 4.1 × 10^−3^ for size 80 (a 16-fold increase), 2.2 × 10^−4^ for size 300 (an 8.5-fold increase) and 5 × 10^−4^ for size 2000 (a twofold increase). However, no change in genetic progress was detected in any of these cases (note the absence of dominance, so there was no potential for inbreeding depression).

## Discussion

This study has shown the differences between the application of genomic and phenotypic selection on genetic progress against hip dysplasia, with genomic selection providing greater genetic progress than the phenotypic scheme using the same parental structure. In the simulated results for the current phenotypic scheme (*r*^2^ = *h*^2^ and testing 33% of each litter at random), the rate of genetic progress achieved per year is very low (ΔTBV = −0.0146), of similar magnitude to that detected by (Lewis *et al*. 2010) for the actual UK Labrador Retriever population. The phenotypic scheme could be improved by maintaining the same number of candidates and using a more restrictive truncation point, but this would lead to an increase in the inbreeding rate, which is also an issue for Labrador breeders. An alternative would be to increase the number of hip-scored animals per litter and keep the truncation point unchanged, which, according to our simulations, would lead to a 2.7-fold increase in genetic gain when scoring 100% of each litter. However, this option is impractical in the current dog breeding culture given the late timing and the risks associated with the scoring method, as most breeding decisions are made when the animals are still puppies and only those animals already chosen for breeding are generally kept and scored. The use of a genomic selection scheme to replace the current phenotypic scheme would solve the difficulties mentioned above, hastening genetic progress by (i) offering an opportunity to increase selection accuracy through a genomic test, (ii) offering an early opportunity to select within litters (the earliness of the test makes this practical) and (iii) removing environmental biases such as age at scoring (Weller *et al*. 2012). The second of these benefits makes use of the additional accuracy of estimating the Mendelian sampling term for an individual, and this also permits greater rates of gain for the same rate of inbreeding (Daetwyler *et al*. 2007).

Previous studies have shown the potential benefits from using BLUP-EBV compared with phenotype alone when selecting against hip dysplasia (Malm *et al*. 2012; Lewis *et al*. 2013). Lewis *et al*. (2010) showed from the analysis of field data that gain in Labrador retrievers could be improved 1.19-fold through accuracy alone when using EBV. In other breeds, increases in the accuracy of EBV compared with phenotypes led to a 1.23-fold improvement in gain on average (Lewis *et al*. 2013). Lewis *et al*. (2010) showed that the mean accuracies of the EBV of sires and dams at the time of breeding of Labrador Retrievers were 0.78 and 0.70, respectively, corresponding to *r*^*2*^ ∼ 0.55. BLUP-EBVs may be considered analogous to simulations with *r*^*2*^ ∼ 0.55 and 33% scored as they offer no early selection opportunities within litters (EBVs of the puppies are the average of their parents until scoring). Interpolating from Table 2, this analogy suggests an estimate of 1.25-fold gain from EBV over phenotypic selection, in good agreement with the estimates from the real data made by Lewis *et al*. (2010, 2013).

Whilst BLUP-EBVs offer worthwhile benefits in increased gain, there are drawbacks compared with genomic selection. First, the naïve use of BLUP may increase rate of inbreeding (Bijma & Woolliams 2000) requiring additional monitoring by the breeding community, whereas genomic selection, as mentioned above, has potential to generate more gain for the same rate of inbreeding (Daetwyler *et al*. 2007). Second, the accuracy of BLUP relies upon continuous recording, and the accuracy is limited by the size and structure of the scheme; in contrast, the accuracy of genomic selection is expected to increase towards 1 as the size of the training data set increases, provided that the SNP chip captures all the genetic variance, with much of the accuracy retained over several generations without retraining. Third, BLUP-EBVs do not offer the possibility of distinguishing between littermates early in life (Daetwyler *et al*. 2007).

Therefore, genomic selection is an attractive possibility but there are problems to overcome before it can be implemented in Labradors. The first of these is developing a training set of Labradors of sufficient size to deliver sufficient accuracy to initiate a scheme. A reasonable benchmark for considering this is the size of training set requited to deliver *r*^*2*^ = 0.50, which in our simulations delivered significant extra gain compared with phenotypic selection and would have the potential to deliver more gain than BLUP-EBV from within-litter testing. Using the deterministic formula of Daetwyler *et al*. (2010) suggests the need for approximately 1500–2000 dogs (assuming an effective population size ∼100). As more dogs obtain genotypes over time, accuracies would be expected to increase. A second problem is the concern that accuracy will deteriorate over time through the decay of linkage disequilibrium between the markers and the causative loci, and predictions would be expected to require regular re-estimation (e.g. Solberg *et al*. 2009). In that study, the decay in accuracy was modelled by the Bulmer effect (Bulmer 1971) which is appropriate for the infinitesimal model, but the actual change in accuracy will depend on the true genetic model as well as factors such as selection intensity and the model used for analysis. However, this decrease would be offset by the accumulation of phenotypic records in the training set and the accompanying re-evaluations. There is risk that breeders would stop scoring once a genomic selection scheme was initiated, but it would be essential to make breeders and their organizations aware that genomic selection requires the re-estimation of marker effects and that more benefits flow from accumulating more data, so that a steady flow of genotyped and phenotyped animals enter the training set.

A further possible drawback to genomic selection is the cost of genotyping using SNP chips. The current cost of the high-density panel and its processing (∼£200) is significant and similar to the cost of phenotypically scoring one animal (£250–500). However, techniques such as imputation from low-density chips have the potential to dramatically reduce costs. In such a scheme, only the widely used sires that make substantial contributions would need to be genotyped with high-density chips, whilst screening would be carried out with much cheaper, low-density chips. The missing data can then be imputed from the pedigree and the high-density data using readily available software (e.g. AlphaImpute; Hickey *et al*. 2011).

The rates of inbreeding obtained in our simulated scenarios and the BVA/KC data set (approximately 1 × 10^−3^ per generation) were lower than the ones reported by Lewis *et al*. (2010) and Calboli *et al*. (2008) (8.2 × 10^−3^ and 4 × 10^−3^ per generation, respectively). The latter estimates were obtained using pedigrees that combined animals both within and outside the hip score scheme, and by contrast, our simulations and actual pedigree analyses were based on the BVA/KC data set, which only contained individuals in the hip score scheme. Within the hip-scoring scheme, only 1 or 2 animals from each litter proceed to be parents and such differences between its management and the wider breed are a plausible explanation for the higher rates of inbreeding observed in the full population compared with the simulated data.

Managing rates of inbreeding are possible using optimum contributions (e.g. Meuwissen 1997; Malm *et al*. 2012) but challenging to implement in the loose co-operatives formed by pedigree dog breeders. The management of inbreeding is outside the scope of this study; however, we have studied the effect of higher inbreeding in the hip score scheme using additional simulated scenarios with lower population sizes. These scenarios led, as expected, to much higher levels of inbreeding, but little change in genetic progress. This is in part due to the fact that inbreeding depression was not incorporated in our simulations, which is consistent with field studies (Mäki 2004), showing minor effects of inbreeding on hip dysplasia. The relative performance of genomic and phenotypic selection was unchanged.

## Conclusions

Using simulations to compare genomic selection with phenotypic selection against hip dysplasia, we have shown that the application of a genomic selection programme offers benefits in gain over the current phenotypic scheme against this disease. The advantage of genomic selection depended on the accuracy achieved and hence on the size of the training set. However, the major benefit of genomic selection arises from the possibility of increasing the number of tested animals from each litter, given the availability of DNA tests at birth which do not carry risks and are compatible with current breeding practices. This increase in the number of animals tested per litter would lead to an increase in the within-litter component of selection, allowing for a 2.2-fold increase in gain when testing 50% of each litter with accuracies of *r*^2^ = 0.5 (*r* = 0.7).
